# An Iron Metabolism-Related Gene Signature for the Prognosis of Colon Cancer

**DOI:** 10.3389/fcell.2021.786684

**Published:** 2022-01-18

**Authors:** Jing Yuan, Tao Liu, Yuhong Zhang

**Affiliations:** State Key Laboratory of Oncology in South China, Collaborative Innovation Center for Cancer Medicine, Sun Yat-sen University Cancer Center, Guangzhou, China

**Keywords:** colon cancer, prognosis, iron metabolism, nomogram model, overall survival

## Abstract

As an essential microelement, the iron ion is involved in cell proliferation, metabolism, and differentiation. Iron metabolism plays a crucial role in the occurrence and development of colon adenocarcinoma (COAD). In this study, univariate and multivariate Cox regression, and least absolute shrinkage and selection operator analyses were conducted to construct the gene signature, based on a dataset from The Cancer Genome Atlas. We identified the prognostic value of two iron metabolism-related genes [SLC39A8 (encoding solute carrier family 39 member 8) and SLC48A1 (encoding solute carrier family 48 member 1)] in COAD. A nomogram model was established to predict the overall survival of patients with COAD. Functional analysis showed that the tumor microenvironment and immune cell infiltrate were different between the low risk and high risk subgroups. This study verified that the iron metabolism-related gene signature (SLC39A8 and SLC48A1) could be used as a prognostic biomarker for patients with COAD.

## Introduction

Colon adenocarcinoma (COAD) is one of the three most commonly diagnosed cancers, and is the second leading cause of cancer death worldwide. Although research enhanced the overall survival and led to good prognosis for patients with COAD, the mortality and disability caused by COAD are still very high (Xi and Xu, 2021).

At present, the biggest challenge to improving the survival of patients with COAD is metastasis or postoperative recurrence (Carethers and Jung, 2015). Therefore, a more accurate prognostic assessment model is required to allow individualized treatment and improve the prognosis of patients with COAD. There is increasing evidence that iron overload is closely associated with tumorigenesis in multiple types of human cancer. Cancers usually have a demand for iron, which is an essential element in biological processes, including DNA synthesis, energy metabolism, and immune function (Behr et al., 2018; Hassannia et al., 2019). Ferroptosis, an iron-dependent form of nonapoptotic cell death, has emerged recently as a novel method to treat cancer (Dixon et al., 2012). Pathways of iron metabolism, including iron acquisition, efflux, storage, and regulation, are unbalanced in cancer, indicating that iron metabolism plays a crucial rules in tumor cell survival (Torti and Torti, 2013). However, the prognostic value of genetic markers associated with iron metabolism in COAD has not been fully explored.

In this study, we constructed iron metabolism-related gene signature [*SLC39A8* (encoding solute carrier family 39 member 8) and *SLC48A1* (encoding solute carrier family 48 member 1)] using a dataset from The Cancer Genome Atlas (TCGA) and validated the stability and reliability of the model in a Gene Expression Omnibus (GEO) dataset. Then, functional enrichment analysis was carried out to determine the potential mechanism of action of the proteins encoded by the two genes. Finally, experiments demonstrated the expression level and function of the two iron metabolism-related genes *in vitro* and *in vivo*.

## Methods

### Data Collection

COAD gene expression profiles and corresponding clinical information were downloaded from the TCGA database (*n* = 512) and GEO database (GSE39582, *n* = 576). Seventy iron metabolism-related genes are listed in Supplementary Table S1, which were derived from a previous study (Wei et al., 2020). The GSE39582 dataset was used to validate the established signature.

### Construction and Validation of an Iron Metabolism-Related Genes Risk Score

The TCGA dataset was used as the training dataset to build the iron metabolism-related genes risk score. Univariable Cox regression and Least absolute shrinkage and selection operator (LASSO) Cox analysis were performed to select iron metabolism-related genes associated with prognosis (*p* < 0.05). Then, multivariate Cox regression was used to further determine candidate iron metabolism-related genes associated with prognosis in COAD. Following this, the risk score for each patient was calculated as follows:

Risk Score=coefficient 1E∗xp1+coefficient 2E∗xp2+.......+coefficient nE∗xp n

where Exp represented the expression level of the candidate iron metabolism-related gene. Using the median of the above risk score as the cutoff point, patients with COAD in the training cohort and validation cohort were divided into low-risk and high-risk subgroups.

### Validation of the Prognostic Model

Kaplan–Meier analysis was carried out to compare the prognostic difference between the two subgroups. Univariable and multivariate Cox regression were conducted to further evaluate the iron metabolism-related gene signature’s prognostic value in the training cohort. Then, the prognostic gene signature was validated externally in the validation dataset, followed by further Kaplan–Meier analysis.

### Constructing a Predictive Nomogram

Univariate and multivariate Cox regression analysis were carried out to determine the prognostic factors including the risk score established above, and age, sex, tumor stage, and tumor-node-metastasis (TNM) stage in COAD. The factors with a significant difference (*p* < 0.05) were selected to construct a nomogram model. A calibration curve was plotted to verify the accuracy of the nomogram.

### Gene Set Enrichment Analysis

Enriched pathways in different iron metabolism risk score datasets were assessed using Gene Set Enrichment Analysis (GSEA) software (GSEAv4.0.1, https://www.gsea-msigdb.org/). The Hallmark (v7.1) gene set collections were used as references. *p* < 0.05 and a false discovery rate of q < 0.25 were considered significant.

### Correlation of the Risk Score With the Proportion of Tumor-Infiltrating Immune Cells

The abundance of 22 TICs in each tumor sample in the COAD cohort were estimated using the CIBERSORT module in the R package. CIBERSORT (Chen et al., 2018) was performed to determine the relative mRNA expression levels in the high- and low-risk subgroups to characterize the cell composition of tumor tissues.

### RNA Isolation and qRT-PCR Analysis

Total RNA from colon cancer cells were extracted using the TRIzol reagent (Takara Biotechnology, Dalian, China). Next, cDNA was prepared using a Revert Aid First Strand cDNA Synthesis kit (Thermo Fisher Scientific, Waltham, MA, United States). The cDNA was then used as a template in a quantitative real-time polymerase chain reaction (qPCR) to determine the expression levels of the iron metabolism-related genes using Japan). The qPCR amplification reactions conditions were as follows: 95°C for 15 min; followed by 40 cycles of 95°C for 30 s, 55°C for 1 min, and 72°C for 30 s. The expression levels were normalized to those of *ACTB* (encoding *β*-actin). All primers were synthesized by Sangon Biotech (Shanghai, China) and are listed in Supplementary Table S2. All PCR reactions were performed in triplicate, and the relative expression levels of mRNA were quantified using the 2-∆∆Ct method.

### Immunohistochemistry Examination

This protocol was approved by the Ethics Committee of Sun Yat-Sen University Cancer Center (Guangzhou, China). Written informed consent was obtained from patients at their first visit. The surgically resected colon cancer tissues of eight patients at Sun Yat-sen University Cancer Center were included in this study. Tumors and corresponding nontumorous tissue were fixed in 4% paraformaldehyde, embedded in paraffin blocks, and processed into 4 µm-thick continuous sections. Immunohistochemical staining was performed to determine the distribution of SLC39A8 and SLC48A1. The antibodies used were: anti-SLC39A8 (1:200; 20459-1-AP; Proteintech) and anti-SLC48A1 (1:200; NBP1-91563; Novus Biologicals).

### Cell Culture and Transfection

Colon cancer cells (DLD-1) were cultured in Dulbecco’s modified Eagle Medium (Gibco, Grand Island, NY, United States) supplemented with 10% fetal bovine serum (Invitrogen, Carlsbad, CA, United States) at 37°C with 5% CO_2_. Small interfering RNAs (siRNAs) targeting *SLC39A8* and *SLC48A1* were synthesized by Sangon Biotech. The sequences of the siRNAs are listed in Supplementary Table S4. Transient transfection was performed using the Lipofectamine 2000 reagent (Invitrogen, Shanghai, China) according to the producer’s protocol.

### Cell Viability Assay

DLD-1 Cells were seeded to a 96-well plate and transfected with negative control (NC)-siRNA, *SLC39A8*-siRNA, or *SLC48A1*-siRNA for 24, 48, or 72 h, after which the cells were further incubated with 20 μL of MTS reagent for another 2 h. Cell viability was detected as the optical density (OD) value at 490 nm.

### Colony Formation Assay

To study the effects of SLC39A8 and SLC48A1 on cell proliferation, DLD-1 Cells transfected with NC-siRNA, *SLC39A8*-siRNA, or *SLC48A1*-siRNA were seeded into 6-well plates and incubated for 14 days. Cells were then stained using Crystal Violet Staining Solution, and the number of colonies was detected using light microscopy.

### Statistical Analysis

All statistical analyses were conducted using the R software. Kaplan–Meier analysis was used to compare the overall survival differences between the low- and high-risk subgroups. Given the possibility of multicollinearity, we used LASSO Cox regression analysis to identify the most valuable prognostic genes among all iron metabolism-related genes. An L1 penalty was set in the LASSO Cox model to shrink some regression coefficients to exactly zero and a 10-fold cross-validation with minimum criteria was performed to find the optimal *λ* value. Univariate and multivariate Cox proportional hazard regression analyses were carried out to evaluate the relationship between the risk score and overall survival. In all instances, a value of *p* < 0.05 was regarded as statistically significant.

## Results

### Identification of Differentially Expressed Iron Metabolism-Related Genes

**FIGURE 1:**
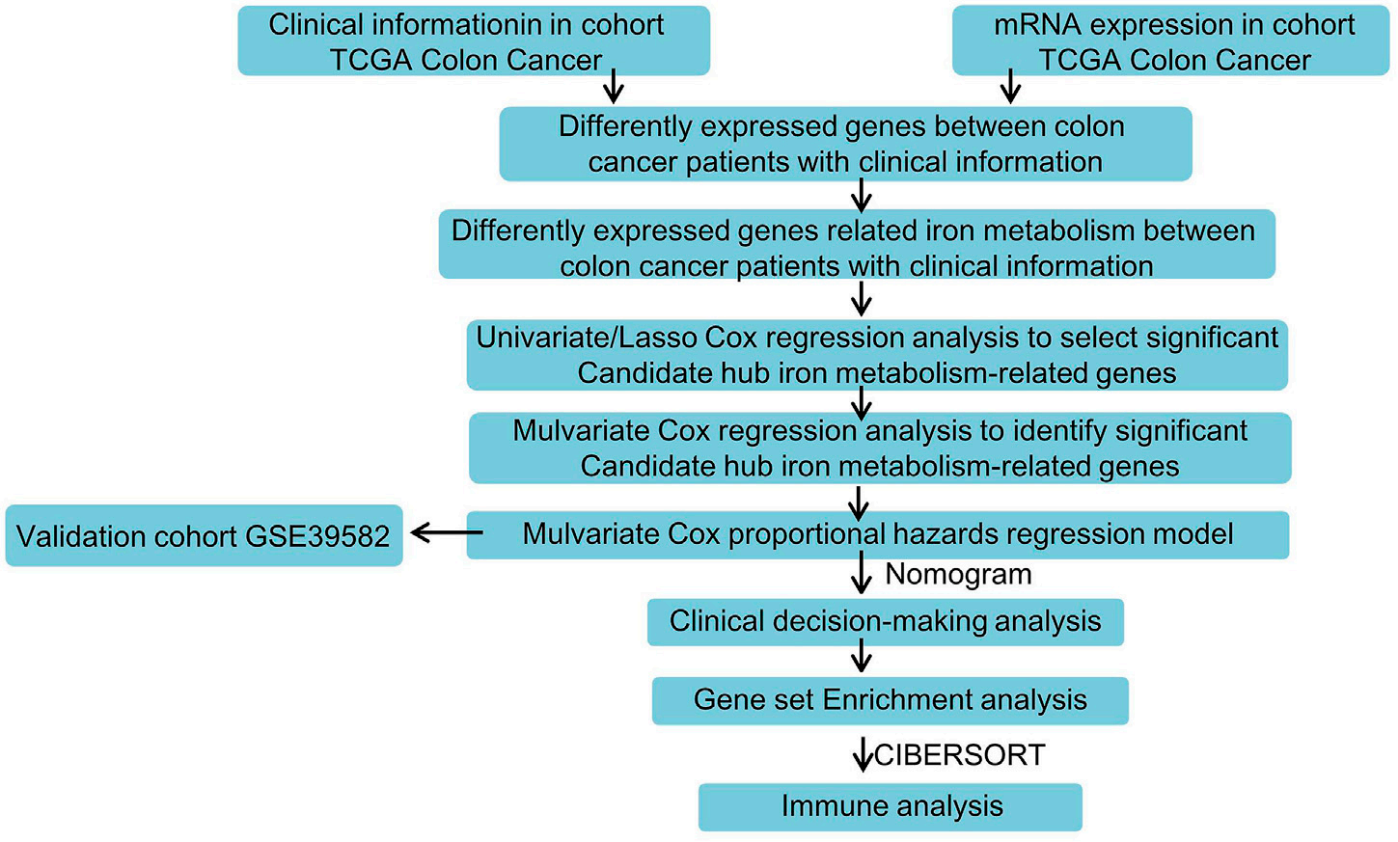
Flowchart for analyzing iron metabolism-related gene signature associated with colon cancer.

As shown in Figure 1, a systematic study was carried out for the pivotal roles and the latent prognostic values of iron metabolism-related genes in COAD. The mRNA expression profiles in tissues from patients with COAD (*n* = 512) were downloaded from the TCGA database as the training dataset. The GSE39582 dataset (*n* = 576) was used as validation cohort. Of the 56,753 genes in the TCGA expression data, 70 iron metabolism-related genes were selected.

### Construction of the Iron Metabolism-Related Gene Signature in the TCGA Cohort

**FIGURE 2:**
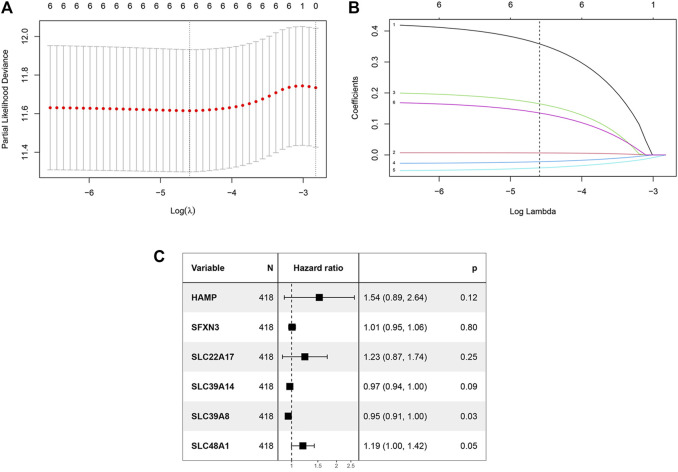
Construction of the iron metabolism model. **(A)** 1000 bootstrap replicates by lasso Cox regression analysis for variable selection. **(B)** LASSO coefficients of iron metabolism genes. Each curve represents a metabolic gene. **(C)** Multivariate Cox regression analysis.

In the TCGA training dataset, single-factor Cox analysis was performed to analyze comprehensively the prognostic value of iron metabolism-related genes in COAD. We found that six genes [*HAMP* (encoding hepcidin antimicrobial peptide), *SFXN3* (encoding sideroflexin 3), *SLC22A17* (encoding solute carrier family 22 member 17), *SLC39A14* (encoding solute carrier family 39 member 14), *SLC39A8*, and *SLC48A1*] were significantly related to the prognosis of patients with COAD (Supplementary Figure S1). These iron metabolism-related genes were subsequently subjected to LASSO Cox regression analysis to avoid colinear influences, and regression coefficients were calculated (Figure 2A). When the six genes were incorporated, the model achieved the best performance (Figure 2B). These genes and their related coefficients are shown in Supplementary Table S3. The six iron metabolism-related genes were further analyzed by multivariate Cox regression. As shown in Figure 2C; Table 1, *SLC39A8* and *SLC48A1* were identified as independent predictors of prognosis for patients with COAD.

**TABLE 1:** Multivariate Cox coefficients of iron metabolism related genes.

Gene	coef	exp (coef)	se (coef)	z	p
HAMP	0.4286	1.535065	0.276562	1.550	0.1212
SFXN3	0.0070	1.007071	0.027600	0.255	0.7985
SLC22A17	0.2046	1.227040	0.176986	1.156	0.2477
SLC39A14	−0.0277	0.972637	0.016133	−1.720	0.0855
SLC39A8	−0.0521	0.949228	0.024044	2.167	0.0302
SLC48A1	0.1739	1.189989	0.089898	1.935	0.0530

### Model Construction and Analysis of the Prognosis-Related Genetic Risk Score

The two hub iron metabolism-related genes identified in *Construction of the Iron Metabolism-Related Gene Signature in the TCGA Cohort* were used to construct a prognosis-related genetic risk score. The risk score of each patient with COAD was calculated as follows:

Risk Score= −0.052106× ExpSLC39A8+0.173944× ExpSLC48A1

**FIGURE 3:**
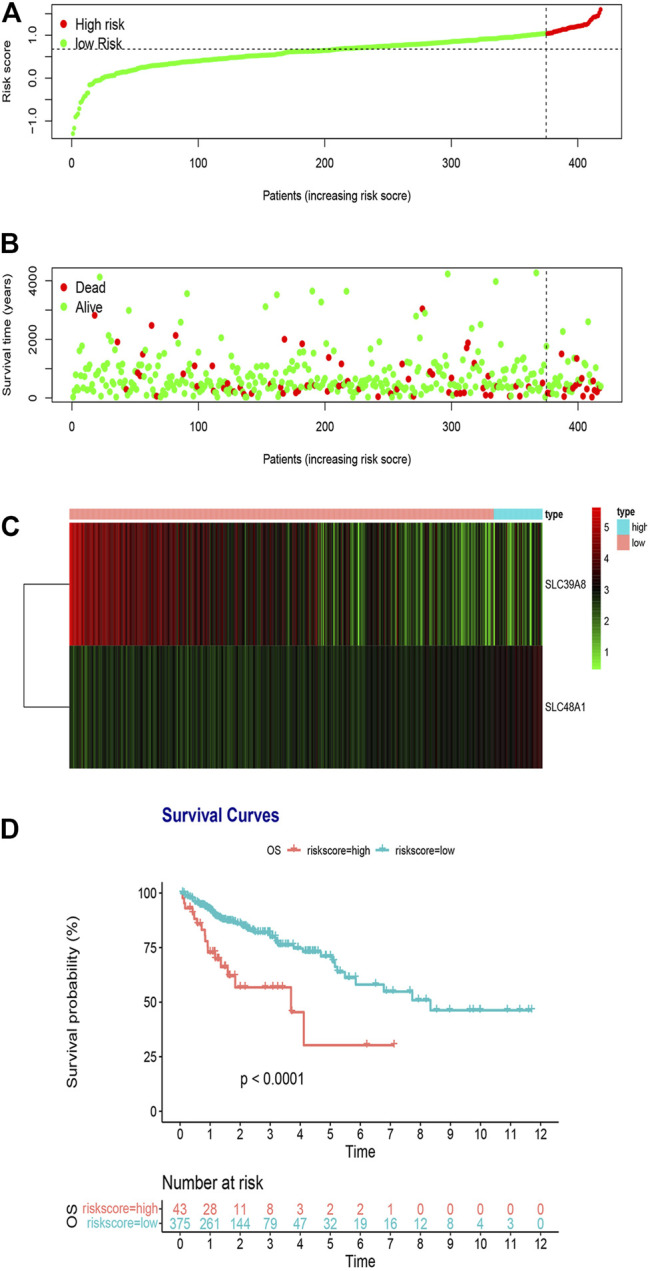
Risk score analysis of the two-gene prognostic model in the TCGA training cohort. **(A)** Survival differences between high- and low-risk groups. **(B)** Dot plots comparing outcomes of subjects in the high- and low-risk groups. **(C)** Heat map for gene expressions in the high- and low-risk groups. **(D)** Kaplan Meier survival analysis of all patients with COAD in the high- and low-risk groups.

We classified risk scores using the optimal cutoff points decided by the maximally selected log-rank statistics, in which patients with risk scores above the cutoff value were recognized as the high-risk group, and patients with risk scores below the cutoff value were recognized as the low-risk group. A survival analysis was performed to evaluate the predictive effect of this model. Figure 3A shows the distribution of the TCGA training cohort. Furthermore, dot plots were made to compare survival of patients in the high-risk and low risk-groups, which showed that survival in the high-risk group was worse than that in the low-risk group (Figure 3B). The heat maps in Figure 3C show the comparison of the expression levels of the two iron metabolism-related genes between the groups. The expression of *SLC39A8* was higher in the low-risk patients, while *SLC48A1* expression was higher in high-risk patients. Kaplan–Meier survival analyses found that patients with COAD in the high-risk group had worse prognosis compared with those in the low-risk group (Figure 3D; *p* < 0.0001).

### Validation of Iron Metabolism-Related Genes Based on the GSE39582, GSE17536, and GSE38832 Dataset

**FIGURE 4:**
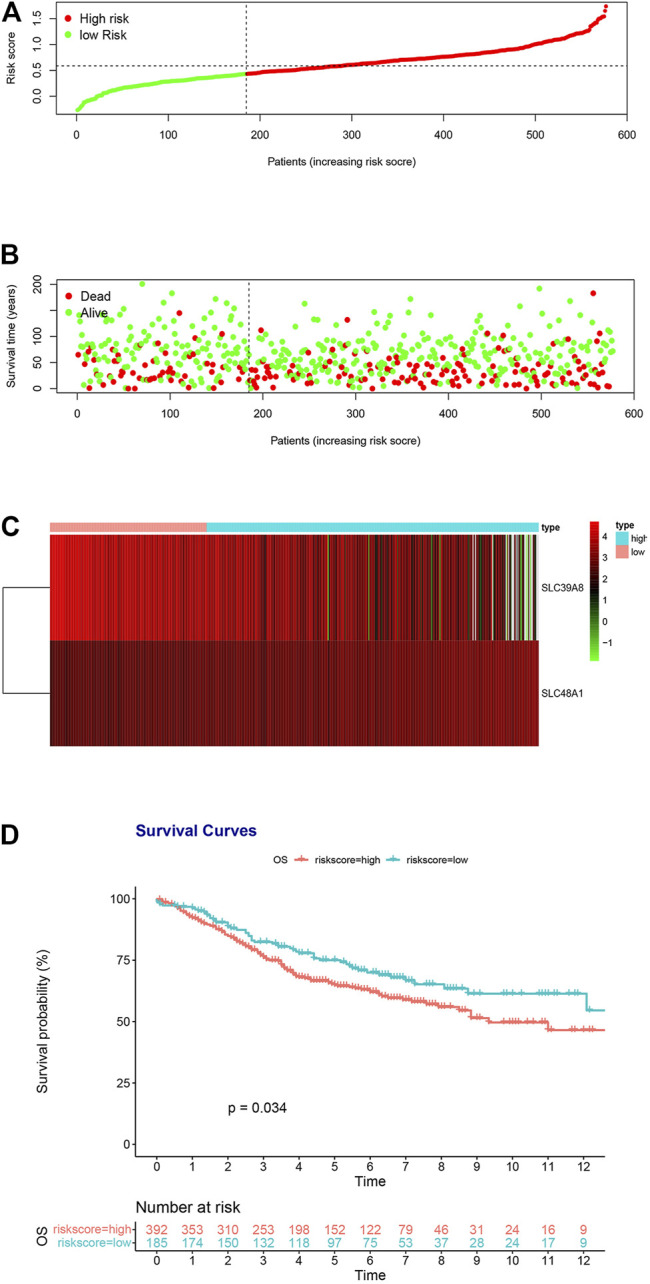
Risk score analysis of the two-gene prognostic model in the GSE39582 validation cohort. **(A)** Survival differences between high- and low-risk groups. **(B)** Dot plots comparing outcomes of subjects in the high- and low-risk groups. **(C)** Heat map for gene expressions in the high- and low-risk groups. **(D)** Kaplan Meier survival analysis of all patients with COAD in the high- and low-risk groups.

To determine the accuracy of the two-gene prognostic model, we used the GSE39582 dataset as an external validation cohort. The distribution of the validation cohort is displayed in Figure 4A, Supplementary Figures S2A, S3A. The dot plot and heat map results were similar to those of the TCGA cohort (Figures 4B,C, Supplementary Figures S2B,C, S3B,C). Survival analysis indicated that the overall survival of the low-risk group was markedly longer than that of the high-risk group (Figure 4D; *p* = 0.034, Supplementary Figure S2D; *p* = 0.053 and Supplementary Figure S3D; *p* = 0.021).

### Development of a Prognostic Model Based on Iron Metabolism-Related Genes and Clinical Factors

**FIGURE 5:**
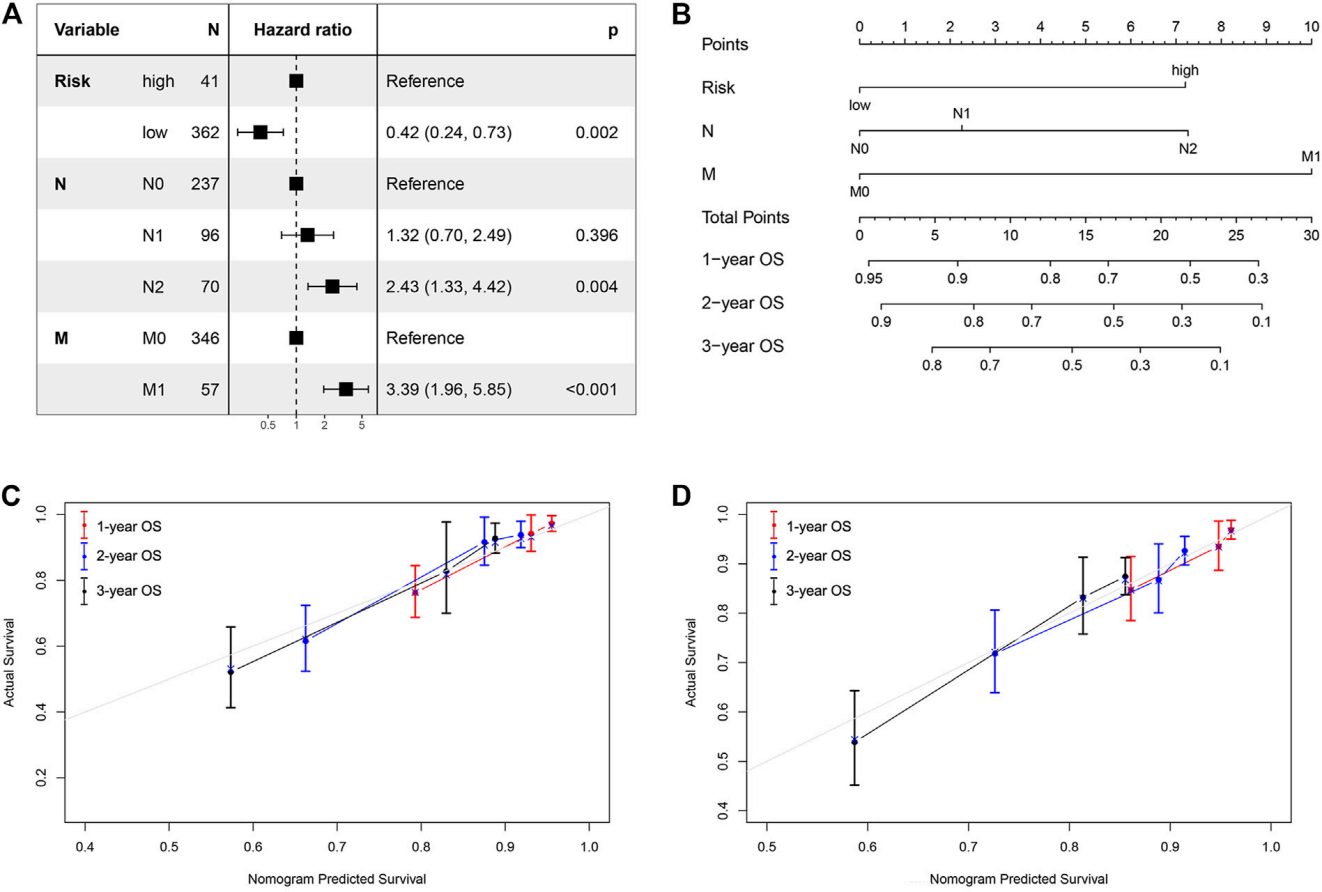
Nomogram and calibration curve for predicting the probability of 1-, 2- and 3-years OS for patients with COAD. **(A)** The hazard ratios of the risk score and clinical factors based on multivariate COX analysis. **(B)** A nomogram integrates iron metabolism gene signature and other prognostic factors in patients with COAD; **(C, D)** The calibration curve of the nomogram in TCGA cohort and GSE39582 validation dataset.

We next analyzed other co-variates using univariate and multivariate Cox regression to determine the prognostic factors in COAD. The results showed that risk score, N stage, and M stage correlated with survival in the multivariate analysis. The hazard ratios of the risk score and above clinical factors are listed in Figure 5A. We then constructed a nomogram using the risk score, N stage, and M stage as variables (Figure 5B). A lower point was related to a better prognostic result on the nomogram. The C-index was 0.8092 [95% confidence interval (CI):0.7543–0.8642] for the nomogram, indicating that it had a good discriminating ability. Outcome was reported as 1-, 2- and 3-years overall survival. The associated calibration curves from the nomograms at 1, 2 and 3 years are displayed in Figures 5C,D, which showed a good performance of our nomogram in predicting 1-, 2- and 3- year survival. Therefore, this nomogram could be a useful model to predict the survival of patients with COAD.

### Gene Set Enrichment Analysis With the Two Iron Metabolism-Related Genes

**FIGURE 6:**
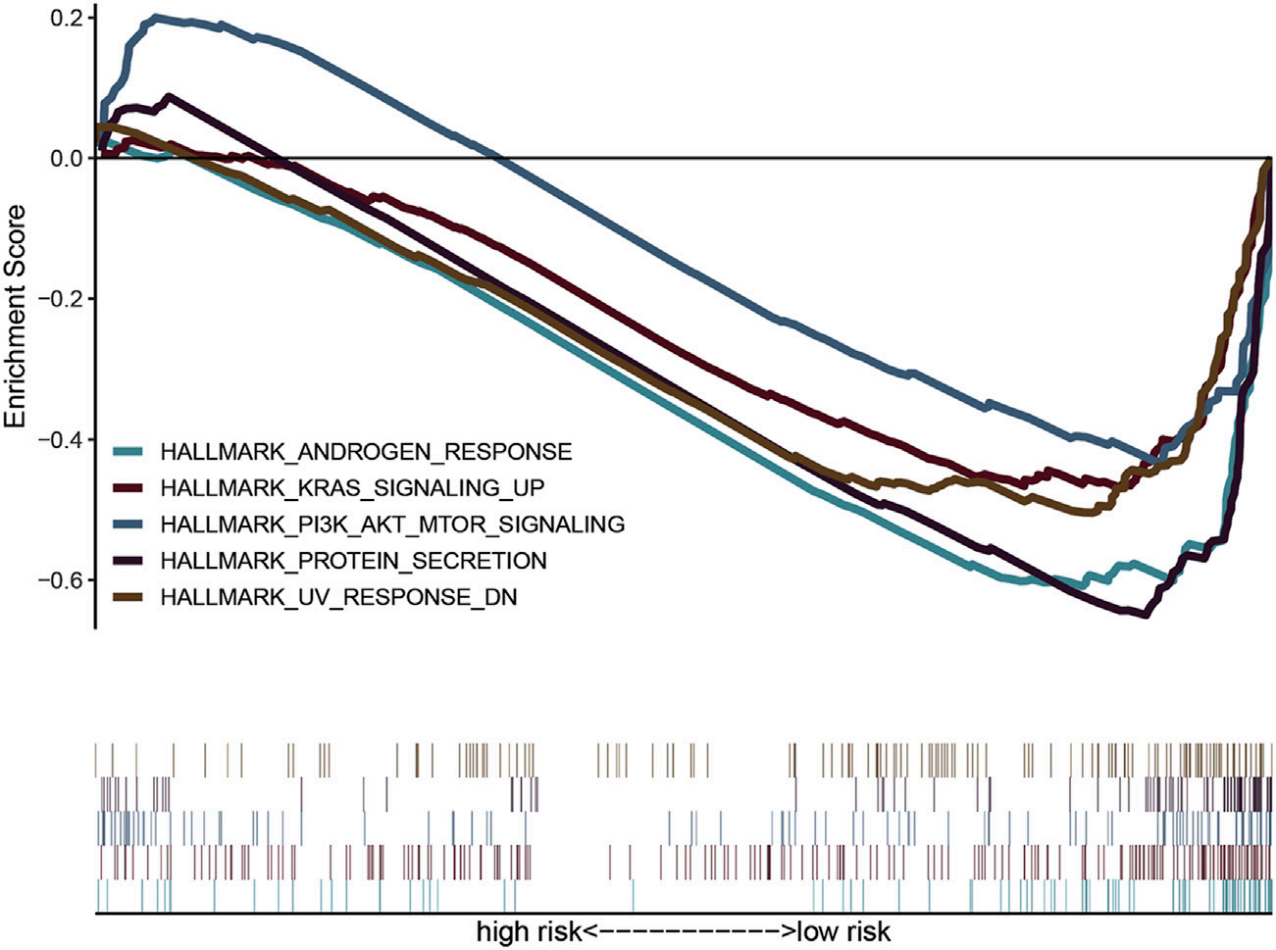
Gene set variation analysis and gene set enrichment analysis of pathways. GSEA showed five pathways enriched in the low-risk group.

To explore the molecular functions of the identified iron metabolism-related genes in this study, we conducted GSEA analysis to determine the gene expression profile. The results are shown in Figure 6. Multiple functional gene sets were enriched significantly in the low-risk group containing HALLMARK_ANDROGEN_RESPONSE, HALLMARK_KRAS_SIGNALING_UP, HALLMARK_PI3K_AKT_MTOR_SIGNALING, HALLMARK_PROTEIN_SECRETION, and HALLMARK_UV_RESPONSE _DN. These findings suggested that the two iron metabolism-related genes were potentially closely correlated with the status of the COAD microenvironment.

### Association of the Two Iron Metabolism-Related Genes With the Proportion of TICs

**FIGURE 7:**
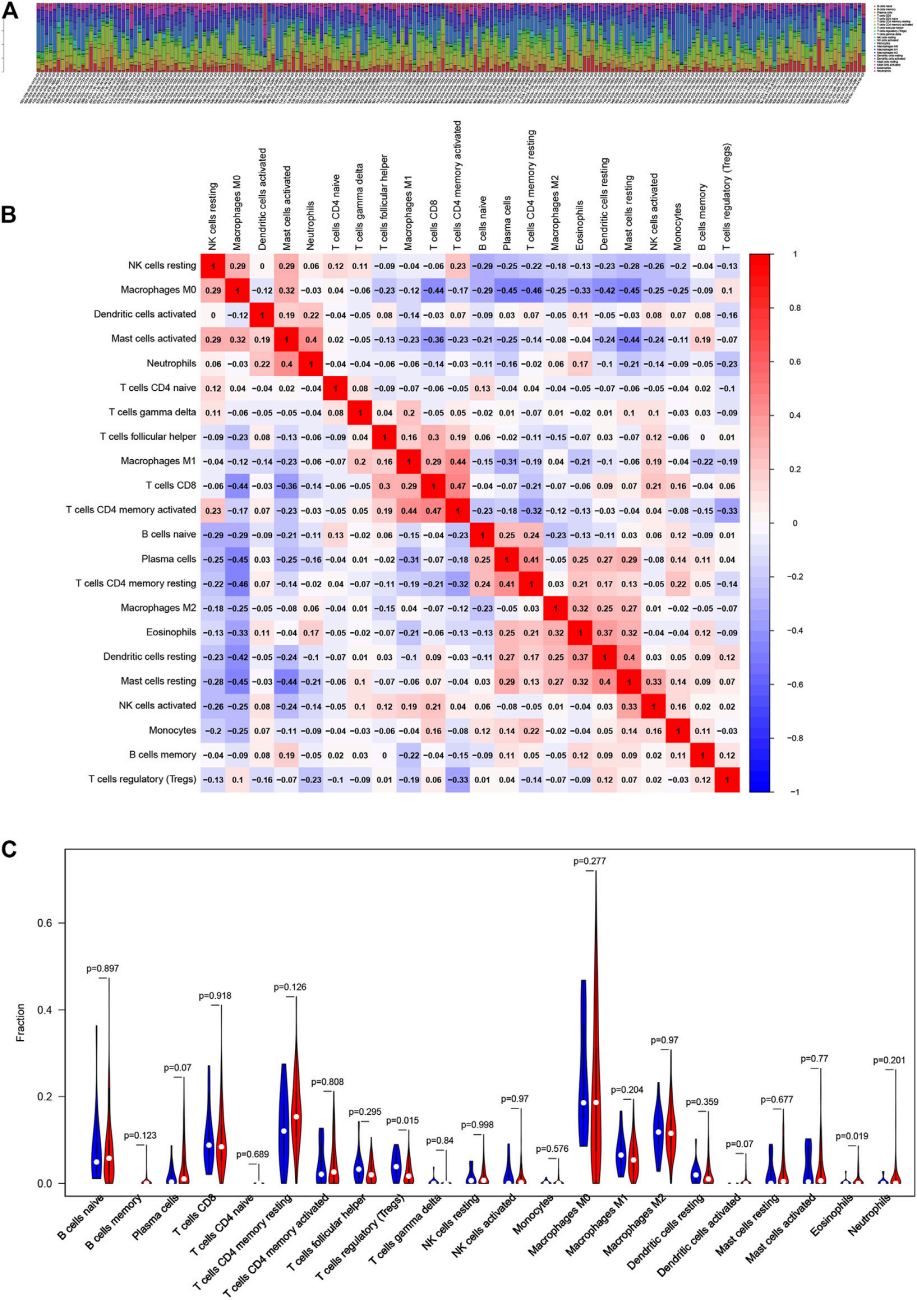
Association of iron metabolism-related gene signature with the proportion of TICs **(A, B)** Bar plot and heat map for the immune microenvironment in the high-risk and low-risk groups **(C)** Distribution level of 22 types of immune cells in the high- and low-risk groups.

To further determine the relationship of the two iron metabolism-related genes with the immune microenvironment, we analyze the proportion of TIC subpopulations and constructed immune cell profiles in COAD using CIBERSORT. As shown in Figures 7A,B, a stacked bar plot and heat map were provided to describe the immune microenvironment in the high-risk and low-risk groups. Furthermore, the proportions of 22 immune cell proportions of COAD are shown in Figure 7C. The results showed that two TICs, regulatory T cells and eosinophils, were related to the two-gene signature risk score. The low- and high-risk score groups showed specific immune cell distributions.

### Expression Level Determination and Functional Analysis of the Iron Metabolism-Related Genes in COAD

**FIGURE 8:**
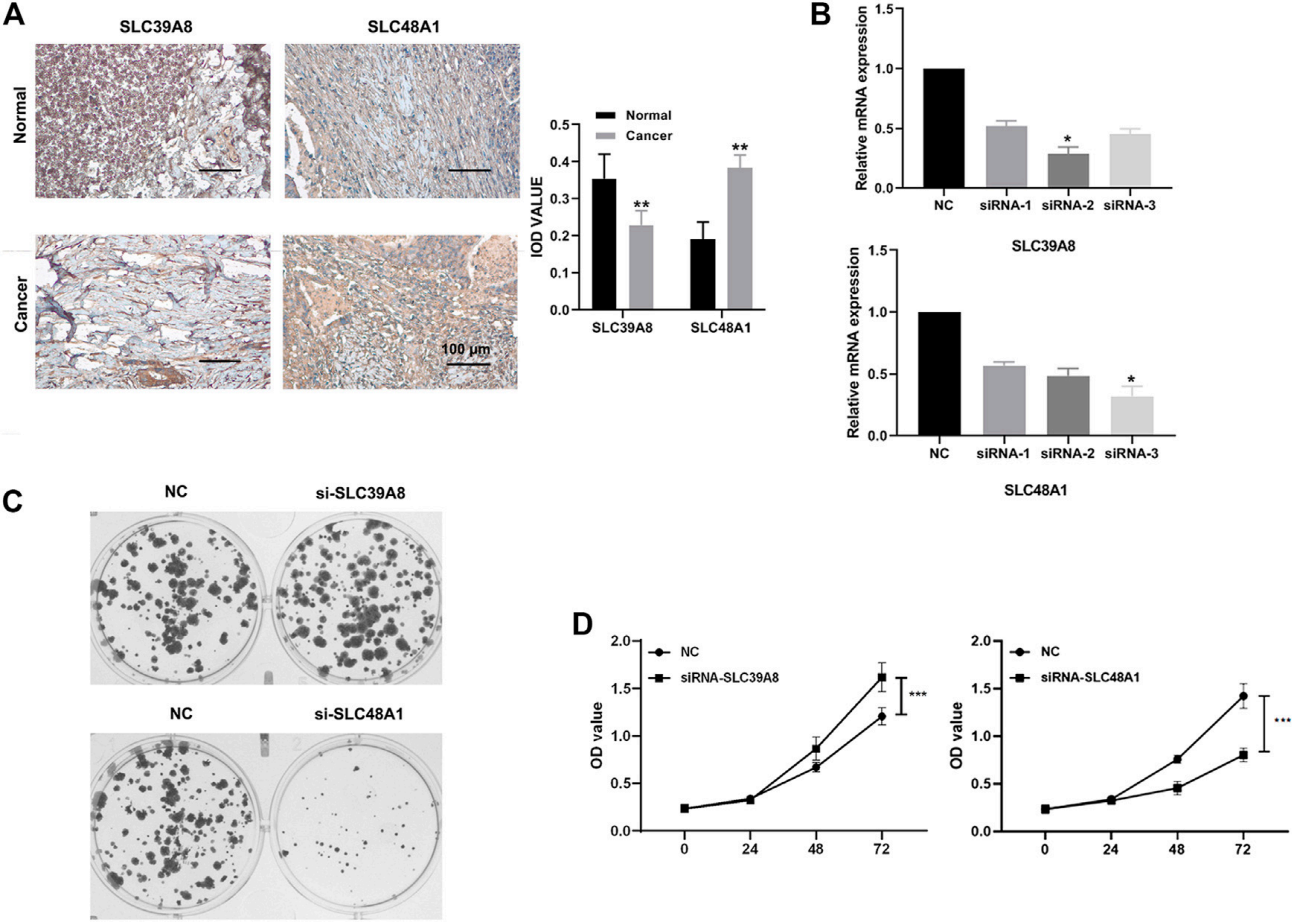
Expression verification and functional analysis of genes related to iron metabolism. **(A)** Immunohistochemical results showed that SLC48A1 was highly expressed in human colon cancer tissues, while SLC39A8 was low expressed in human colon cancer tissues. **(B)** mRNA levels of SLC39A8 and SLC48A1 were knocked down using siRNAs in colon cancer cells. The results of cell clonal formation assay **(C)** and MTS **(D)** showed that knockdown SLC48A1 inhibited the proliferation of DLD1 cells, while knockdown SLC39A8 promoted the proliferation of DLD1 cells. Data are shown as mean ± SD; *p < 0.05, ***p < 0.001 (versus NC group).

To ascertain the expression levels of SLC39A8 and SLC48A1 in COAD tissues, eight colon cancer tissues and corresponding normal tissues were tested. Immunohistochemistry (Figure 8A) showed that the SLC39A8 level was downregulated in colon cancer tissues, and the SLC48A1 level was upregulated significantly in colon cancer tissues. Furthermore, the mRNA levels of *SLC39A8* and *SLC48A1* were successfully knocked down using siRNA in colon cancer cells *in vitro* (Figure 8B, Supplementary Figure S4). Next, we analyzed the potential function of SLC39A8 and SLC48A1 in colon cancer. Knocking down *SLC39A8* promoted the proliferation of different colon cancer cell lines (DLD1, HCT116, and HT29), and silencing *SLC48A1* suppressed the proliferation of colon cancer cells *in vitro* (Figures 8C,D, Supplementary Figure S4). These results indicated that *SLC39A8* might function as a tumor suppressor gene and *SLC48A1* might function as an oncogene in COAD.

## Discussion

The reasons for the occurrence and development of colon cancer are complex and may include interactions between environmental exposure, diet, and genetic factors (Brenner et al., 2014). In the pathogenesis of colon cancer, there are also many genetic and epigenetic changes in proliferation signaling pathways and tumor suppressor genes, such as the WNT pathway, the transforming growth factor beta (TGF-β) pathway, the phosphatidylinositol-4,5-bisphosphate 3-kinase (PI3K)-protein kinase (AKT) pathway, the mitogen activated protein kinase (MAPK) pathway, and the tumor protein p53 (TP53) pathway. Traditional TNM staging has some limitations for accurate prognostic prediction (Sjoblom et al., 2006). Although many molecular markers related to the prognosis of colon cancer have been reported, a single prognostic factor is often one-sided in the precision treatment system, whether it is a traditional pathological indicator or a new molecular marker. Studies have confirmed that the combination of molecular markers and traditional pathological prognostic indicators can predict the prognosis of patients with tumors more accurately. Therefore, in the construction of a prognosis prediction system for colon cancer, researchers have focused on integrating different types of prognostic factors to achieve the goal of predicting patient prognosis accurately.

Iron is an essential metal micronutrient for humans. At the cellular level, iron is involved in basic energy metabolism, mitochondrial function, and DNA synthesis (Imam et al., 2017). At the systemic level, iron metabolism is mainly regulated by the liver-derived endocrine hormone, ferrimodulin. Epidemiological data suggest that iron levels are associated with the risk of colorectal cancer. People with a high intake of red meat containing high amounts of heme iron and patients with iron overload disease had an increased risk of colorectal cancer (Martin et al., 2018). Colorectal cancer cells are enriched in iron relative to adjacent normal intestinal epithelial cells. Iron plays an important role in colorectal cancer. The tumor hypoxic environment induces heterotopic high expression of iron modulin in colorectal cancer epithelial cells through hypoxia-inducible factor 2 alpha (HIF2α) (Schwartz et al., 2021).

In this study, we downloaded data from the TCGA and GEO from public databases and extracted information about 70 genes related to iron metabolism. Univariate regression analysis, multivariate Cox regression analysis, and LASSO Cox analysis were used to identify the genes associated with iron metabolism prognosis in the TCGA cohort, and LASSO Cox regression analysis was used to establish the prognostic model incorporating these genes. We identified two iron metabolism genes, *SLC48A1* and *SLC39A8*, which were associated with clinical survival. In the TCGA and GSE39582 datasets, genes related to iron metabolism can be used to predict the prognosis of patients with COAD. In different datasets of patients with colon cancer, *SLC48A1* and *SLC39A8* gene markers showed good prognostic performance.

The stability of the prediction model was verified in the GEO cohort, and a Nomogram model was constructed to predict the prognosis of patients with COAD. At the same time, the correlation of the *SLC48A1* and *SLC39A8* genes with tumor-infiltrating immune cells was analyzed. SLC48A1 regulates V-ATPase activity, which is a prerequisite for endosomal acidification, and enhances glucose transporter-1 (GLUT-1) transport, while increasing glucose uptake and lactate production. SLC48A1 also promotes the transport of the insulin-like growth factor I receptor (IGF-1R) (Fogarty et al., 2014). SLC39A8 is widely expressed and encodes the zrt- and irt-like protein 8 (ZIP8) protein. ZIP8 (also known as solute carrier family 39 member 8) is a membrane transporter that helps to absorb many substrates, including basic and toxic divalent metals (e.g., zinc, manganese, iron, and cadmium) and inorganic selenium (Liu et al., 2018). We found that high expression of *SLC39A8* and *SLC48A1* in patients with COAD was closely associated with reduced overall survival. Additionally, high expression of SLC48A1 drives glycolysis flux and promotes cancer cell growth, migration, and invasion, which is associated with poor prognosis (Sohoni et al., 2019). The loss of ZIP8 inhibits the migration potential of neuroblastoma cancer cells by reducing the expression level of matrix metalloproteinases (Mei et al., 2018). In this study, we revealed the prognostic value of iron metabolism-related genes *SLC48A1* and *SLC39A8* in colon cancer; however, their associated signaling pathways need to be further explored.

Univariate and multivariate Cox analyses in our training set (TCGA) and validation set (GSE39582) showed that iron metabolism-related genes were independent prognostic factors in patients with COAD. Colon cancer, including COAD, is insensitive to immunotherapy, which might be mediated by a series of immune escape mechanisms. To better understand the relationship between the risk score and immune components, we investigated the association of genes related to iron metabolism with various immune-infiltrating cells. The results showed that the a high risk score was closely related to regulatory T cells and eosinophils. This suggested that the poor prognosis in the high-risk group might be caused by immunosuppression induced by regulatory T cells and eosinophils. Thus, the clinical prognosis of patients with colon cancer may be related to differences in immune cell compositions and genes related to iron metabolism might be involved in immunosuppression in colon cancer. This will provide new insights and targets for immunotherapy of colon cancer.

We used GSEA enrichment analysis to better understand the pathway of iron metabolism-related genes in colon cancer. The results showed that low-risk scores were enriched in KRAS and PI3K-AKT-mechanistic target of rapamycin (mTOR) signaling pathways. The KRAS subtype is mutated in 84% of RAS mutated cancers. In colon cancer, *KRAS* mutations are present in 30–50% of patients (Goel et al., 2015). The presence of *KRAS* mutations not only affects prognostic survival, but also predicts the responsiveness of patients with colon cancer to epidermal growth factor receptor (EGFR) signaling inhibitors (Stec et al., 2012). Overexpression of PI3K/AKT/mTOR signaling components has been reported in various types of cancer, and is especially closely related to the occurrence, development, and prognosis of colon cancer. In recent years, inhibitors targeting PI3K/AKT signaling have been shown to reduce the tumor burden in different experimental models and have been considered as potential therapeutic agents (Johnson et al., 2010; Narayanankutty, 2019). The possibility that iron metabolism might function through KRAS and PI3K-AKT-mTOR signaling pathways in colon cancer provides ideas for future research.

IHC was used to further verify the clinical samples, which showed high SLC39A8 levels in tumor tissues. Iron is known to be essential for the catalytic function of ribonucleotide reductase, the enzyme that converts ribonucleotides into deoxyribonucleotides, a rate-limiting step in DNA synthesis and an obligate step in cell replication (Torti and Torti, 2020). Moreover, iron was also proved to promote the proliferation and Warburg Effect of colon cancer cells through colony formation and MTS experiment *in vitro* (Yuan et al., 2021). HIF-2α promoted cell proliferation and survival by inducing iron accumulation in HCT116 cells, and low-iron diet reduced HIF-2α-mediated intestinal tumorigenesis and cellular proliferation (Xue et al., 2012). As we all know, cell colony formation assay and MTS assay are important techniques for detecting cell proliferation, invasiveness and sensitivity to killing factors. Therefore, we conducted colony formation and MTS experiment to determine the effect of knocking down iron metabolism-related genes on cell proliferation ability in different colorectal cancer cell lines, and the result showed that inhibition of SLC48A1 promoted the proliferation of colon cancer cells, and silencing of SLC39A8 expression suppressed the proliferation of colon cancer cells *in vitro.* These studies also suggest that SLC48A1 and SLC39A8 might be potential predictors of clinical prognosis and therapeutic targets for colon cancer.

Compared with markers based on single gene expression, multi-gene markers obtained by univariate and multivariate Cox and LASSO regression analysis of gene sets can compensate for individual differences, improve prediction and accuracy in tumors, and show better predictive performance. The mechanisms of action of the proteins encoded by iron metabolism-related genes in colon cancer require further study. Meanwhile, although we have noted correlations with immune cells, the function of iron metabolism in the tumor microenvironment and immunotherapy still needs to be clarified.

## Conclusion

A novel iron metabolism-related gene signature based model was constructed that could be used for prognostic prediction in colon cancer. SLC39A8 and SLC48A1 play a role in the development of colon cancer and might be potential therapeutic targets.

## Data Availability

The datasets presented in this study can be found in online repositories. The names of the repository/repositories and accession number(s) can be found in the article/Supplementary Material.
