# Supplementary material for: An Iron Metabolism-Related Gene Signature for the Prognosis of Colon Cancer
Source: Front Cell Dev Biol. 2022 Jan 18;9:786684. doi: 10.3389/fcell.2021.786684 (PMC8804292; doi:10.3389/fcell.2021.786684)
Supplement: Supplementary file 1 [file DataSheet1.PDF]

Supplementary Table 1. Iron metabolism related genes

ID	Ensembl	Description
NUBP1	ENSG00000103274	nucleotide binding protein 1
FBXL5	ENSG00000118564	F-box and leucine rich repeat protein 5
TMEM199	ENSG00000244045	transmembrane protein 199
HJV	ENSG00000168509	hemojuvelin BMP co-receptor
ALAS1	ENSG00000023330	5'-aminolevulinate synthase 1
ALAS2	ENSG00000158578	5'-aminolevulinate synthase 2
ISCU	ENSG00000136003	iron-sulfur cluster assembly enzyme
SLC22A17	ENSG00000092096	solute carrier family 22 member 17
FXN	ENSG00000165060	frataxin
GLRX3	ENSG00000108010	glutaredoxin 3
SLC40A1	ENSG00000138449	solute carrier family 40 member 1
ABCB8	ENSG00000197150	ATP binding cassette subfamily B member 8
BDH2	ENSG00000164039	3-hydroxybutyrate dehydrogenase 2
BMP6	ENSG00000153162	bone morphogenetic protein 6
CP	ENSG00000047457	ceruloplasmin
HEPH	ENSG00000089472	hephaestin
LTF	ENSG00000012223	lactotransferrin
NDFIP1	ENSG00000131507	Nedd4 family interacting protein 1
LCN2	ENSG00000148346	lipocalin 2
HAVCR1	ENSG00000113249	hepatitis A virus cellular receptor 1
SCARA5	ENSG00000168079	scavenger receptor class A member 5
FLVCR1	ENSG00000162769	feline leukemia virus subgroup C cellular receptor 1
ABCG2	ENSG00000118777	ATP binding cassette subfamily G member 2
CYBRD1	ENSG00000071967	cytochrome b reductase 1
SLC48A1	ENSG00000211584	solute carrier family 48 member 1
SLC46A1	ENSG00000076351	solute carrier family 46 member 1
LRP1	ENSG00000123384	LDL receptor related protein 1
CD163	ENSG00000177575	CD163 molecule
FLVCR2	ENSG00000119686	feline leukemia virus subgroup C cellular receptor family member 2
SLC39A14	ENSG00000104635	solute carrier family 39 member 14
SLC39A8	ENSG00000138821	solute carrier family 39 member 8
SLC11A2	ENSG00000110911	solute carrier family 11 member 2
SLC25A37	ENSG00000147454	solute carrier family 25 member 37
HPX	ENSG00000110169	hemopexin
ERFE	ENSG00000178752	erythroferrone
TMPRSS6	ENSG00000187045	transmembrane serine protease 6
HAMP	ENSG00000105697	hepcidin antimicrobial peptide
SLC11A1	ENSG00000018280	solute carrier family 11 member 1
EIF2AK1	ENSG00000086232	eukaryotic translation initiation factor 2 alpha kinase 1
STEAP2	ENSG00000157214	STEAP2 metalloreductase

HFE	ENSG00000010704	homeostatic iron regulator
CUBN	ENSG00000107611	cubilin
TFRC	ENSG00000072274	transferrin receptor
TFR2	ENSG00000106327	transferrin receptor 2
HMOX1	ENSG00000100292	heme oxygenase 1
HMOX2	ENSG00000103415	heme oxygenase 2
STEAP1	ENSG00000164647	STEAP family member 1
STEAP3	ENSG00000115107	STEAP3 metalloreductase
STEAP4	ENSG00000127954	STEAP4 metalloreductase
ABCB7	ENSG00000131269	ATP binding cassette subfamily B member 7
ABCB10	ENSG00000135776	ATP binding cassette subfamily B member 10
ABCB6	ENSG00000115657	ATP binding cassette subfamily B member 6
SFXN1	ENSG00000164466	sideroflexin 1
SFXN2	ENSG00000156398	sideroflexin 2
SFXN3	ENSG00000107819	sideroflexin 3
SFXN4	ENSG00000183605	sideroflexin 4
SFXN5	ENSG00000144040	sideroflexin 5
ATP6AP1	ENSG00000071553	ATPase H+ transporting accessory protein 1
SLC25A28	ENSG00000155287	solute carrier family 25 member 28
ACO1	ENSG00000122729	aconitase 1
IREB2	ENSG00000136381	iron responsive element binding protein 2
FTH1	ENSG00000167996	ferritin heavy chain 1
FTL	ENSG00000087086	ferritin light chain
NCOA4	ENSG00000266412	nuclear receptor coactivator 4
PCBP1	ENSG00000169564	poly(rC) binding protein 1
PCBP2	ENSG00000197111	poly(rC) binding protein 2
FTHL17	ENSG00000132446	ferritin heavy chain like 17
TF	ENSG00000091513	transferrin
FTMT	ENSG00000181867	ferritin mitochondrial
FECH	ENSG00000066926	ferrochelatase

Supplementary Table 2. Primer sequences for qRT-PCR

Gene	Primer Sequence
SLC39A8	Forward: TGTGACATGCTATGCAAATCCT
	Reverse: CCTGTAGAGATACCACACTGACA
SLC48A1	Forward: CGTCCCCTTTCGAGGAAACC
	Reverse: GCGTGCTAAGAAAACAGATCCAG

Supplementary Table 3. LASSO coefficients of iron metabolism related genes

Gene	Coef
HAMP	0.358365764611003
SFXN3	0.00650952131679285
SLC22A17	0.165064512889558
SLC39A14	-0.0218633256786092
SLC39A8	-0.0418545264153228
SLC48A1	0.135386421456954

Supplementary Table 4. The sequences of siRNA-SLC48A1 and siRNA-SLC39A8

Gene	Primer Sequence
siRNA -SLC48A1_001	GCACGTGATGTACATGCAA
siRNA -SLC48A1_002	GGAGCTTCATTTCCTTCAA
siRNA -SLC48A1_003	GGGCTGACTTTGCTGACAT
siRNA -SLC39A8_001	GGTGTGACATGCTATGCAA
siRNA -SLC39A8_002	GGGACTCAGTACTTCCATA
siRNA -SLC39A8_003	CCAGCAGTCTTACAGCAAT

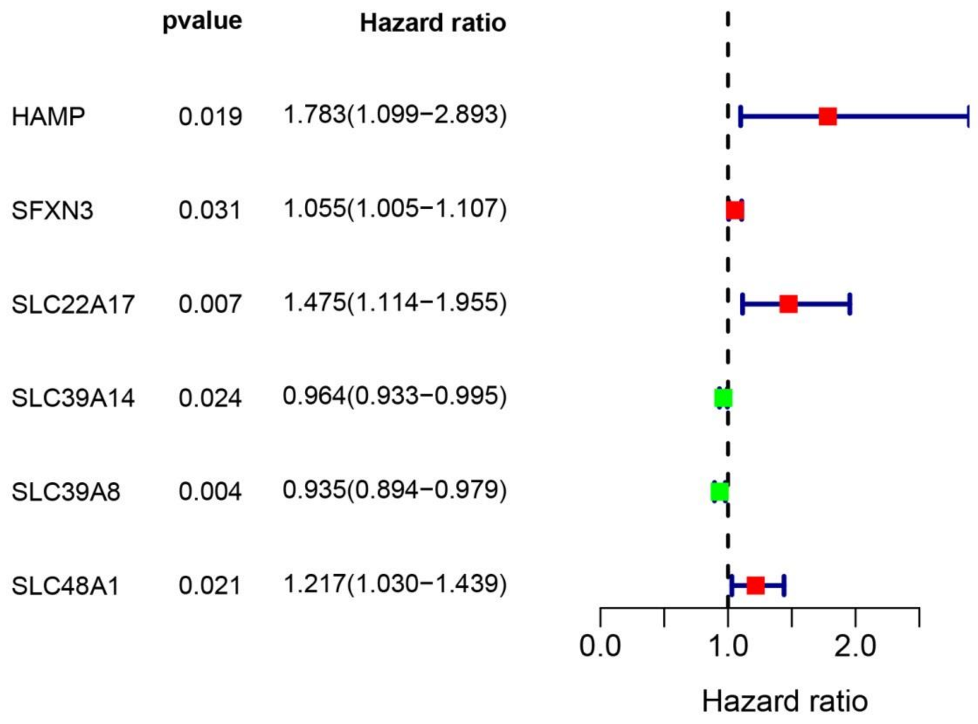

Figure S1 Identification of prognosis-associated iron metabolism-related genes

according to Univariate Cox regression analysis.

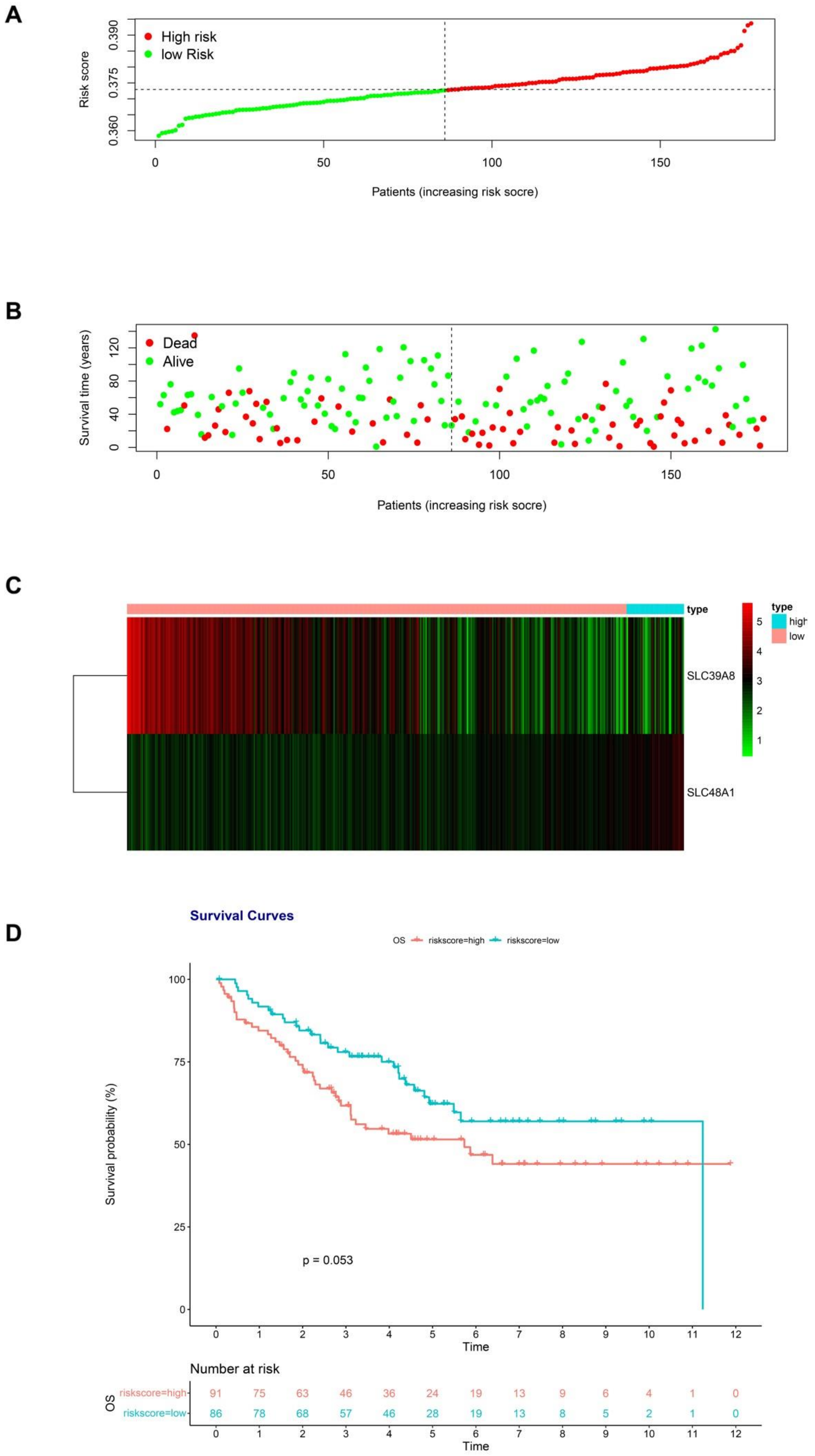
A
High risk
low Risk
Risk score
0.360
0.375
0.390
0
50
100
150
Patients (increasing risk socre)
B
Dead
Alive
Survival time (years)
0
40
80
120
0
50
100
150
Patients (increasing risk socre)
C
type
high
low
5
4
3
2
1
SLC39A8
SLC48A1
D
Survival Curves
OS
riskscore=high
riskscore=low
Survival probability (%)
100
75
50
25
0
p = 0.053
0 1 2 3 4 5 6 7 8 9 10 11 12
Time
Number at risk
OS
riskscore=high 91 75 63 46 36 24 19 13 9 6 4 1 0
riskscore=low 86 78 68 57 46 28 19 13 8 5 2 1 0
0 1 2 3 4 5 6 7 8 9 10 11 12
Time

Figure S2 Risk score analysis of the two-gene prognostic model in the GSE17536 validation cohort. (A) Survival differences between high- and low-risk groups. (B) Dot plots comparing outcomes of subjects in the high- and low-risk groups. (C) Heat map for gene expressions in the high- and low-risk groups. (D) Kaplan Meier survival analysis of all patients with COAD in the high- and low-risk groups.

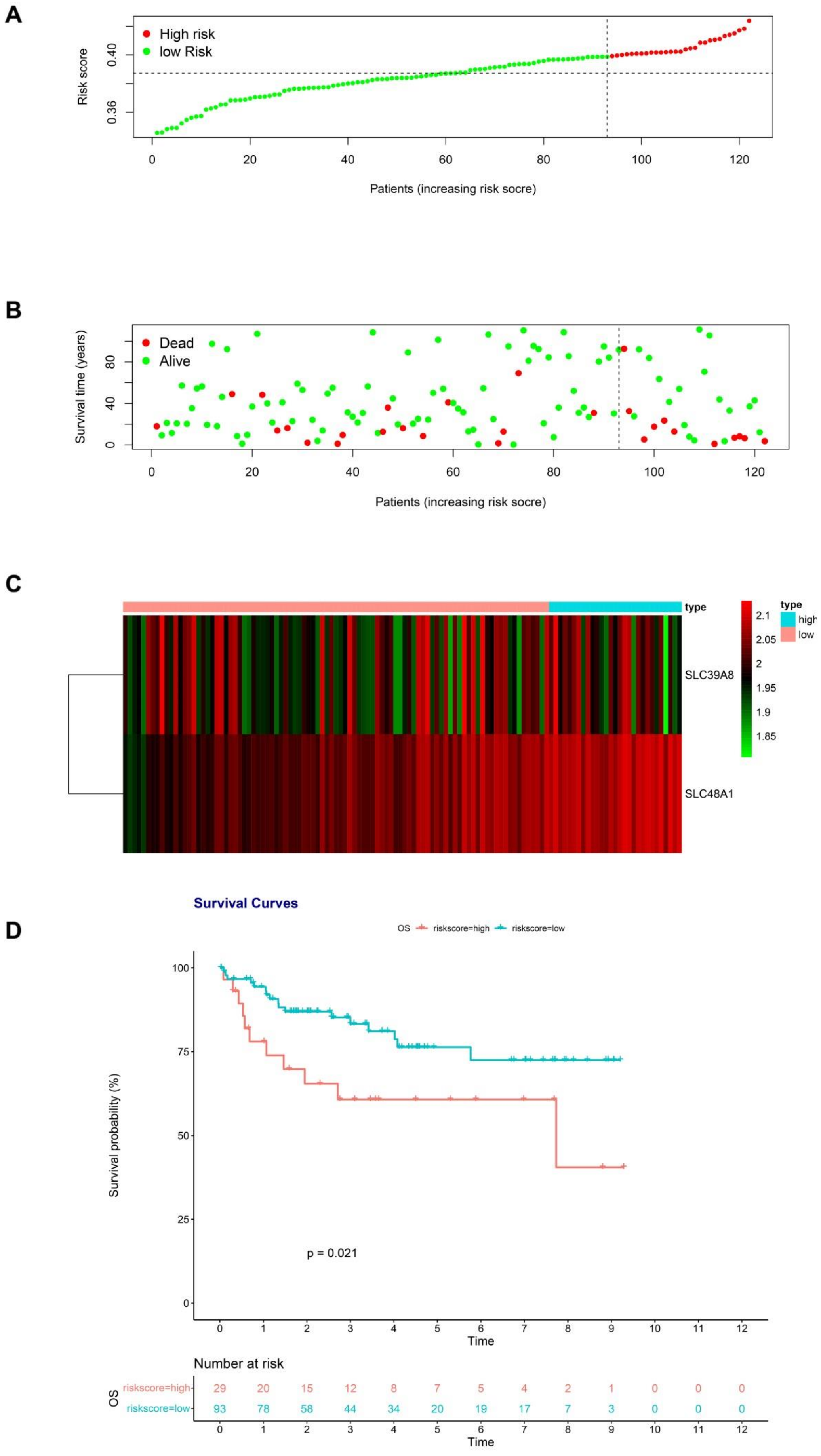
A
High risk
low Risk
Risk score
Patients (increasing risk socre)
B
Dead
Alive
Survival time (years)
Patients (increasing risk socre)
C
type
high
low
SLC39A8
SLC48A1
D
Survival Curves
OS
riskscore=high
riskscore=low
Survival probability (%)
p = 0.021
Time
Number at risk
riskscore=high 29 20 15 12 8 7 5 4 2 1 0 0 0
riskscore=low 93 78 58 44 34 20 19 17 7 3 0 0 0

Figure S3 Risk score analysis of the two-gene prognostic model in the GSE38832 validation cohort. (A) Survival differences between high- and low-risk groups. (B) Dot plots comparing outcomes of subjects in the high- and low-risk groups. (C) Heat map for gene expressions in the high- and low-risk groups. (D) Kaplan Meier survival analysis of all patients with COAD in the high- and low-risk groups.

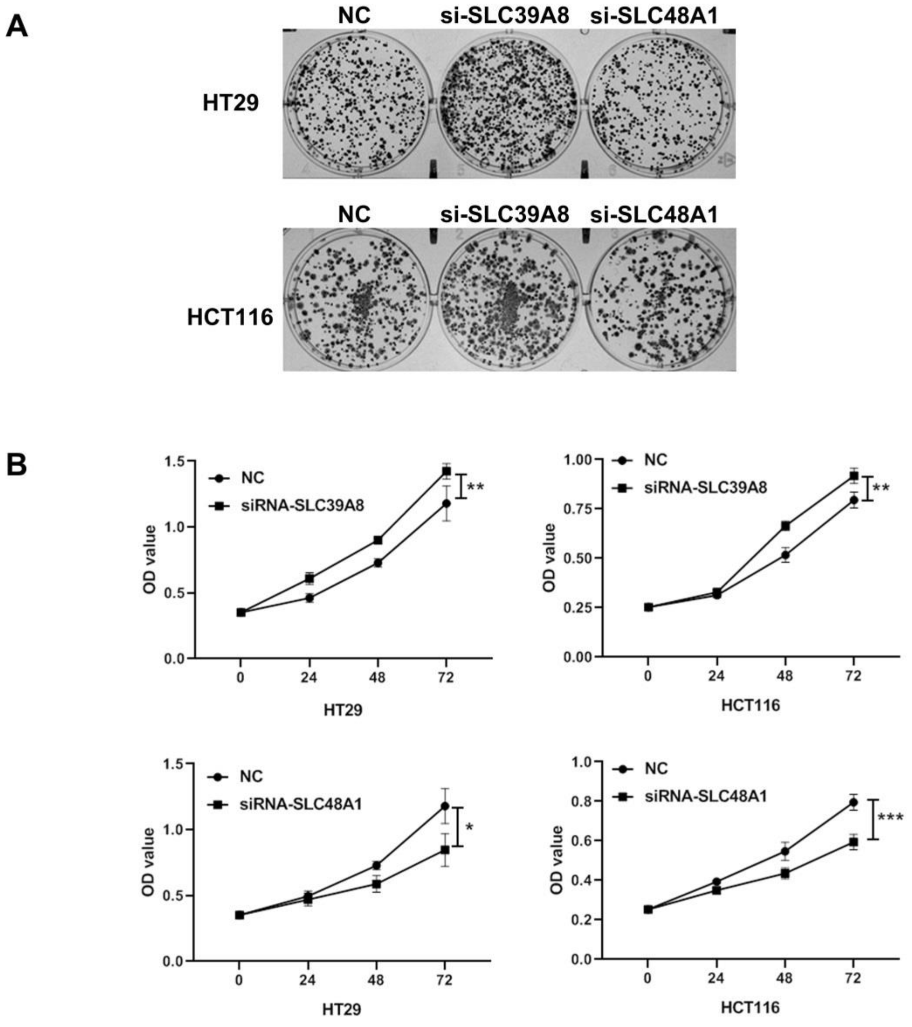

FigureS4 The phenotypes of SLC39A8 and SLC48A1 in different colon cancer cell lines. The results of cell clonal formation assay (A) and MTS (B) showed that knockdown SLC48A1 inhibited the proliferation of HCT116 and HT29 cells, while knockdown of SLC39A8 promoted cell growth in HCT116 and HT29 cells. Data are shown as mean ± SD; *P < 0.05, **P < 0.01, ***P < 0.001 (versus NC group).

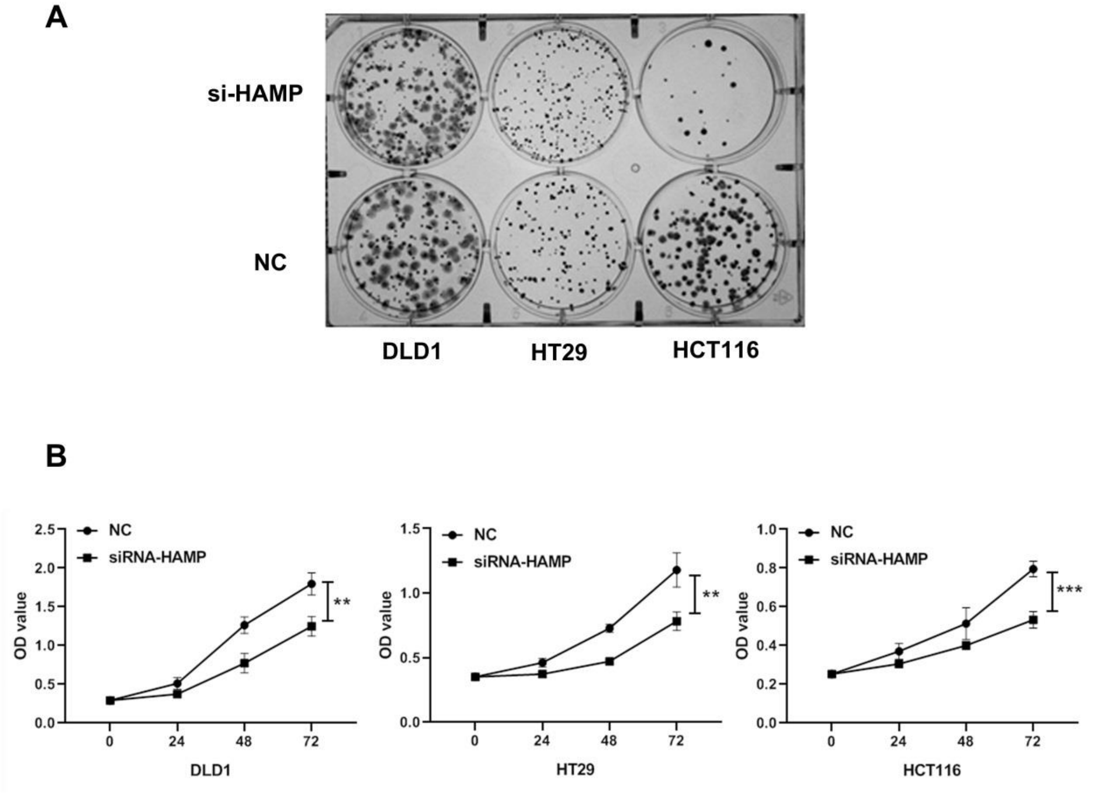

FigureS5 Knockdown of HAMP inhibited the growth of colorectal cancer cells.The results of cell clonal formation assay (A) and MTS (B) showed that knockdown of HAMP inhibited the proliferation of DL1,HCT116 and HT29 cells, Data are shown as mean ± SD; **P < 0.05, ***P < 0.001 (versus NC group).
